# Feast or famine: embedding a specialist clinic in a tertiary child and youth mental health service

**DOI:** 10.1186/2050-2974-2-S1-O15

**Published:** 2014-11-24

**Authors:** Tania Withington, Richard Litster, Salvatore Catania, Judi Krause

**Affiliations:** 1 Child and Youth Mental Health Service, Queensland Children's Hospital, Brisbane, Australia

## 

Anorexia Nervosa is a high cost low prevalence eating disorder in children and young people presenting to tertiary child and youth mental health services across the country. The disorder creates high anxiety amongst service providers as it usually requires long term high resource service provision with limited recovery rates. Current evidence based practice identifies Family Based Treatment as the most effective treatment option with the highest rates of recovery sustained over time, however the success of the intervention has been linked with clinical practice holding model integrity. Current evidence indicates that best outcomes are achieved when delivery of treatment is provided through specialist Eating Disorders services.

This presentation will share the journey of embedding a specialist multi-disciplinary eating disorder clinic utilising Family Based Treatment, within the limits of existing resources, in a large metropolitan Hospital and Health Service in Queensland. Challenges including freeing resources from other service areas, culture and practice shifts in the treatment of eating disorders, managing competing interests in the treatment of eating disorders, developing partnerships across internal and external stakeholders, and, building a sustainable workforce and service model will be discussed. Future directions including research, establishing a comprehensive evaluation strategy, accreditation of clinicians as Family Based Treatment specialists, provision of training and supervision, and seeking funding to establish an independent service will be outlined.

This abstract was presented in the **Service Initiatives: Child and Adolescent Refeeding and FBT** stream of the 2014 ANZAED Conference.

